# Food-Borne Polycyclic Aromatic Hydrocarbons and Circadian Disruption

**DOI:** 10.1021/acsomega.4c04120

**Published:** 2024-07-09

**Authors:** Yen-Chun Koh, Min-Hsiung Pan

**Affiliations:** † Institute of Food Science and Technology, National Taiwan University, Taipei 106017, Taiwan; ‡ Department of Medical Research, China Medical University Hospital, China Medical University, Taichung City 404327, Taiwan; § Department of Health and Nutrition Biotechnology, Asia University, Taichung City 413305, Taiwan

## Abstract

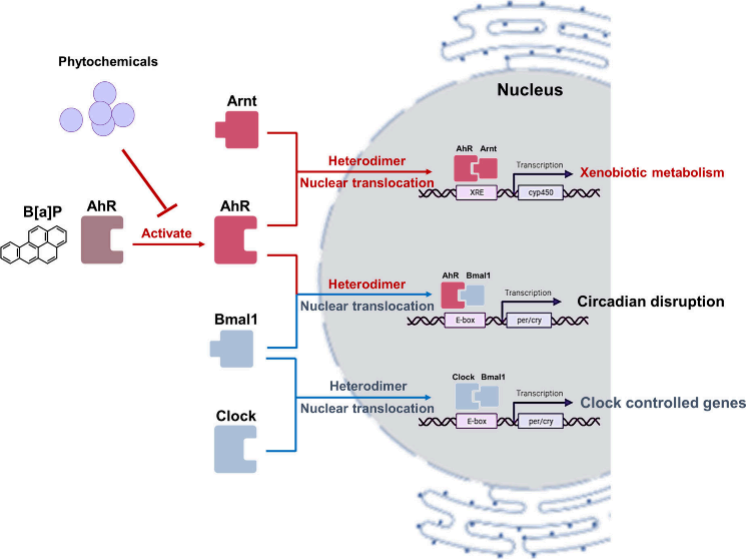

Circadian disruption has been found to increase the risk of metabolic diseases, brain disorders, and cancer. The aryl hydrocarbon receptor (AhR), responsible for xenobiotic metabolism, is known to be activated by certain environmental stimuli, including polycyclic aromatic hydrocarbons (PAHs). Exposure to these stimuli may lead to diseases related to circadian disruption, with AhR activation suggested as a leading cause. Both the aryl hydrocarbon receptor nuclear translocator (ARNT) and aryl hydrocarbon receptor nuclear translocator-like (BMAL1) are class II basic helix–loop–helix/Per-ARNT-SIM (bHLH-PAS) proteins. These proteins form heterodimers with stimulated class I bHLH-PAS proteins, including circadian locomotor output cycles kaput (CLOCK) and AhR. Due to their sequential similarity, the overactivation of AhR by toxicants, such as PAHs, may lead to the formation of heterodimers with BMAL1, potentially causing circadian disruption. Dysregulation of BMAL1 can affect a wide range of metabolic genes, emphasizing its crucial roles. However, this issue has not been adequately addressed. Previous studies have reported that the inhibitory effects of phytochemicals on AhR activation can ameliorate diseases induced by environmental toxicants. Additionally, some phytochemicals have shown preventive effects on circadian misalignment. Therefore, this Review aims to explore potential strategies to prevent circadian disruption induced by food-borne toxicants, such as benzo[*a*]pyrene; to generate new ideas for future studies; and to highlight the importance of investigating these preventive strategies.

## 1 Circadian Clock

Research on the mechanisms controlling circadian rhythms was awarded the 2017 Nobel Prize in Physiology and Medicine; this provided the concept that rhythmic outputs of expression of some genes are regulated by clock genes, including aryl hydrocarbon receptor nuclear translocator-like (*BMAL1*), circadian locomotor output cycles kaput (*Clock*), cryptochrome (*Crys*), and period (*Pers*), which form a regulatory feedback loop controlled by signals in a cell-autonomous and self-sustained manner.^1,2^ Thousands of genes are driven by these core circadian regulators and result in tissue-specific functions under a certain rhythmicity.^3^

### 1.1 Circadian Clock, Master Clock, and Peripheral Clock

The circadian clock serves as the internal timing machine that ensures organisms maintain the rhythmic consistency to adapt to the Earth’s rotational period of around 24 h.^4^ The rhythmic biological clock can autonomously regulate the above-mentioned physiological functions of organisms periodically in the short-term absence of environmental cues, such as light.^5^ Most mammals present regular rhythmic oscillation, known as circadian rhythm, in their regular activities such as the sleep–wake cycle and dietary stress, which regulate essential physiological functions including body temperature maintenance, metabolism processes, energy homeostasis, and hormone secretion.^4^ The endogenous biological rhythm, approximately 24 h in duration, controls many biological functions, such as gene expression, and is preserved in most organisms across evolution.^6^

**Figure 1:**
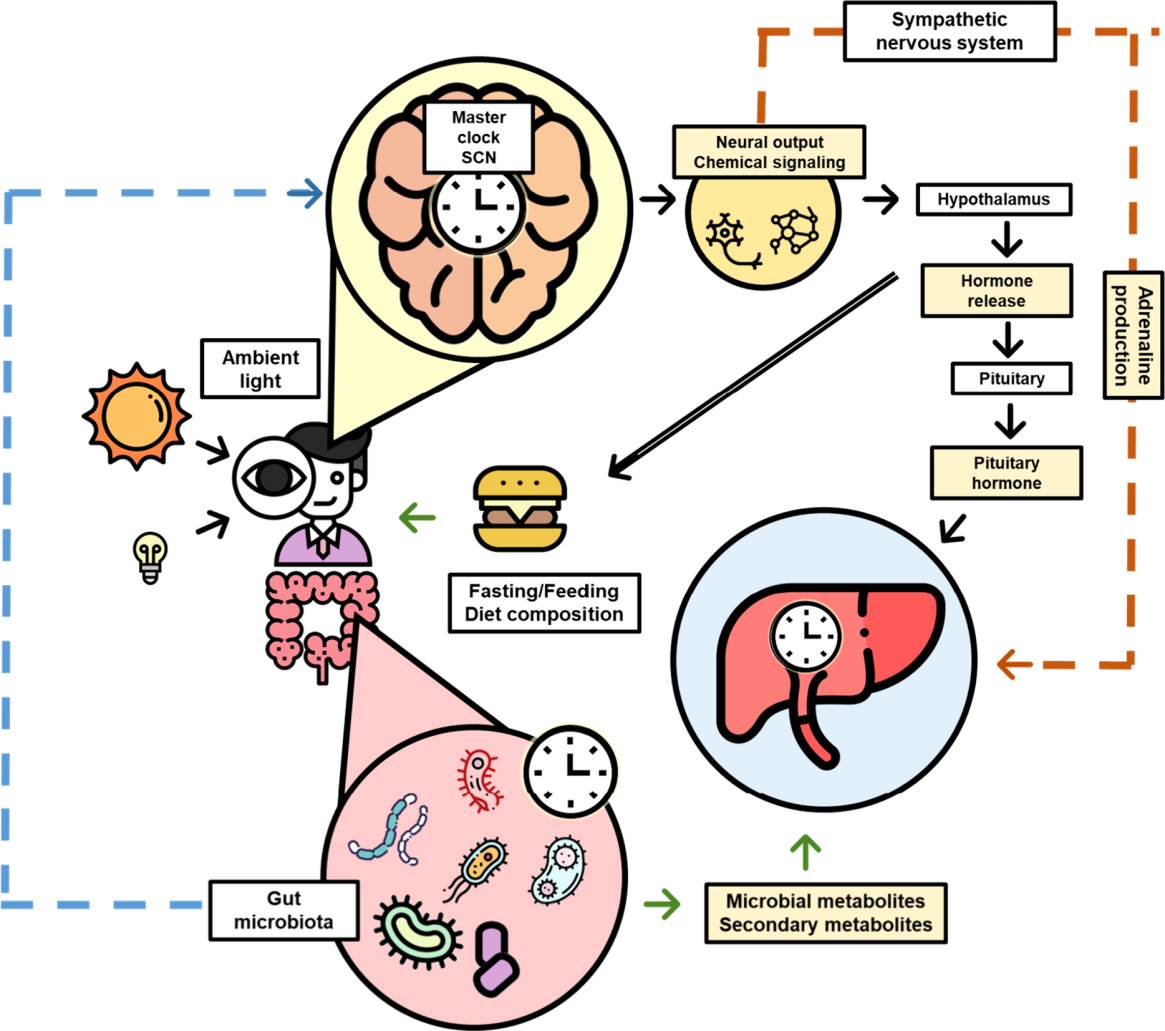
Overview of the principal circadian clock of mammals. Ambient light is one of the entrainment factors that affect the master clock and hormone output, and these hormone signals will control the peripheral clocks in other organs, thereby impacting fasting/feeding times. Fasting/feeding times and diet composition are also entrainment factors that could regulate peripheral clocks and the compositions of gut microbiota. Consequently, gut microbial metabolites affect peripheral clocks in organs such as the liver.

The suprachiasmatic nucleus (SCN), the central pacemaker of the circadian clock known as the master clock of the mammalian hypothalamus, is responsible for modifying the rhythmic output signals with light stimuli.^7^ It is recognized as the dominant controller of behavioral rhythm. However, circadian genes are capable of expressing in isolated peripheral organs with or without signals from the SCN.^4^ Light is known as the only exogenous photic entrainment to the mammalian circadian system, but there are other nonphotic entrainments that hold biological significance.^8^ Notably, light and nutrient consumption have a large impact on biological rhythms, and the latter could directly affect peripherals (Figure 1).^9^ “There is no organ and no function in the body which does not exhibit a similar daily rhythmicity”, according to Gierse (1842) and restated by Dr. Aschoff (1965). The concept that circadian rhythm affects the body’s functionality and peripheral liver has been proposed for at least nine decades.^10^

**Figure 2:**
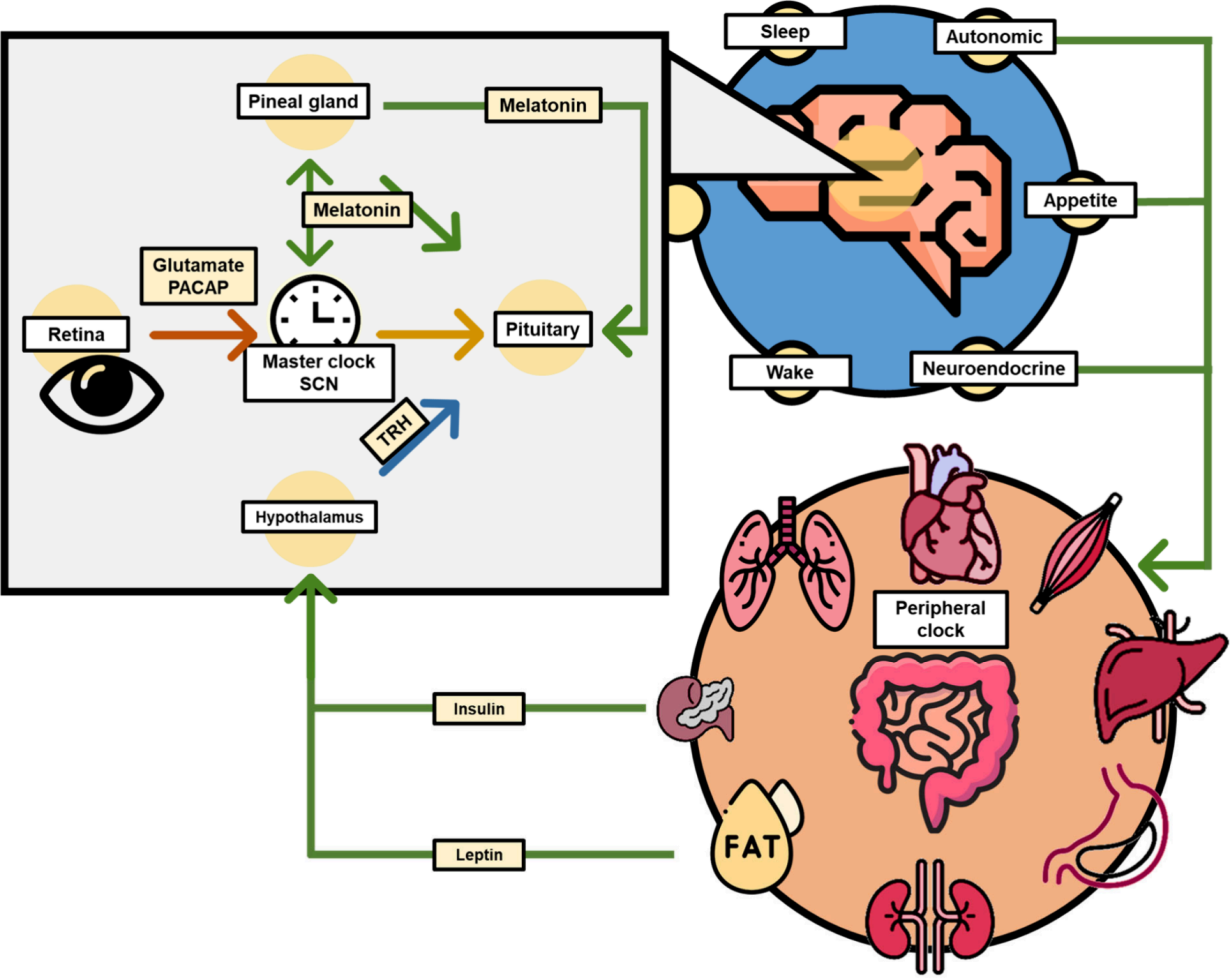
The principal circadian clock of mammals via hormone secretion. The peripheral clocks of organs affected by hormones controlled by the circadian clock include the lung, heart, muscle, liver, stomach, kidney, adipose tissue, and pancreas. Hormones secreted by these tissues could provide feedback to the master clock.

Nonphotic entrainment could be partially responsible for circadian misalignment. Unlike the master clock SCN, the circadian rhythm of peripheral clocks in other organs or tissues can be potentially disrupted by a variety of environmental stimulative factors including but not limited to temperature, jet lag, sleep disorder, dietary stress, artificial light, and stress causing allostatic load accumulation.^11,12^ The circadian activities in different tissues can be specifically modulated by certain signaling molecules produced by neuroendocrine tissues and their interaction with their cognate receptor counterparts located and functioning in those tissues.^9^ Briefly, via endocrine outflow, the SCN clock could affect organ function dependently/independently through peripheral clock synchronization (Figure 2).^13^ Furthermore, exposure to environmental toxicants and drugs has been shown to have a disruptive impact on circadian rhythm, which can result in metabolic disease development.^14^

This is indicated in the growing epidemiological evidence that shows shift work has become a risk factor for hypertension, stroke, and coronary heart disease, while social jetlag may be associated with an elevation in triglyceride levels, a reduction in high-density lipoprotein (HDL), and insulin sensitivity.^15^ Cardiovascular disease, nonalcoholic fatty liver disease (NAFLD),^16^ obesity, and leptin resistance^17^ are the suggested consequences of metabolic dysfunction due to circadian disruption. These results are supported by established findings that provide evidence that energy homeostasis, especially glucose and lipid metabolism, could be regulated by circadian machinery.^14^ Moreover, disruptive effects on circadian rhythm may also lead to cognitive deficits and neurodegenerative diseases, such as Alzheimer’s disease and Parkinson’s disease.^9^ Mutation or knocking out of clock genes such as BMAL1, Per2, Cry1, and Cry2 in animal models has revealed their regulatory impacts on mood behaviors.^11^

### 1.2 Molecular Mechanism

It is clearly understood that the molecular clock of most mammals constitutes a transcriptional–translational feedback loop (TTFL) that consists of core clock genes including initiators, *Clock*, brain-and-muscle ARNT-like protein (*BMAL1*) that transcribes “period” genes (*PER1*, *PER2*, and *PER3*), cryptochrome genes (*CRY1* and *CRY2*) that function as inhibitors in the transcriptional feedback loop, and Rev-Erb-α and ROR-α that can positively and negatively regulate *BMAL1.*(11) In addition to core clock genes, the binding of heterodimer CLOCK-BMAL1 onto the E-box located in the promoter region also promotes the transcriptome. It is suggested that 7–13% of genes are regulated by the circadian system, so-called clock-controlled genes (CCGs). Although the core components of molecular clocks are mostly the same in different tissues, the tissue-specific expression patterns of these circadian genes lead to minorly overlapped cycling transcripts.^2^ As listed by Zhang and Kay in 2010, some CCGs have been successfully identified in which their clock function and their oscillations controlled by the intrinsic clock are responsible for the efficiency of energy use and environmental change anticipation.^18^ These CCGs encode proteins involved in transcription, translation, signal transduction, the cell cycle, and metabolic processes that have been widely discussed in recent years.

In the following section, the role of BMAL1, which has been identified as a factor in the incidence of diseases or malfunction of organs, will be introduced.

#### 1.2.1 Brain and Muscle ARNT-like Protein (BMAL1)

Brain and muscle ARNT-like protein-1 (BMAL1) is a protein that is expressed in some brain regions at a high level and is responsible for the synchronization of peripheral oscillations in muscle and other organs.^19^ As a major and essential component of the circadian clock, BMAL1 is one of the most widely studied genes because of its impact on disease incidence. In 2008, Lamia et al. found significant physiological changes in liver-specific deleted *BMAL1*^*–/–*^ mice in both normal light–dark cycles and constant dark.^20^ Under the conditions of a standard light–dark cycle, *BMAL1*^*–/–*^ mice showed significant increases in body weight and total fat content, slower restoration of blood glucose, and hypersensitivity trends in serum insulin. At the molecular level, the absence of BMAL1 led to a loss of the rhythmic expression of *glucose transporter 2* (*Glut2*) in both mRNA and protein levels. Deletion of *BMAL1* in the liver was found to be more relevant to metabolic dysregulation, while mutation of *BMAL1* (*Mop3*) nonspecifically in certain organs may increase the progression of aging and mortality. In 2005, Burger et al. found the development of joint ankyloses in *Mop3* null mice, which severely decreased their activity levels.^21^ It was concluded that the absence of the MOP3 protein was the major factor causing mice to exhibit reduced locomotor activity and fail to thrive during aging due to abnormal ectopic mineralization and ossification at the insertion site of tissues and bones. Therefore, *Mop3* was suggested to be a potential inhibitor of ossification in ligaments and tendons (Table 1).

**Table 1:** Changes in or the Impact of BMAL1 on the Phenotype Observed

cell line/animal species	gene and type of invalidation	impact on molecular changes	impact on phenotype	ref
C57BL/6 × 129	liver-specific *BMAL1*^*–/–*^	loss of circadian expression in *Glut2*	at the age between 4 and 8 weeks: rapidly gained weight, increased total fat content, glucose intolerance, insulin hypersensitivity	(20)
			at 14 weeks of age: progressive arthropathy	
C57BL/6J (gene targeting in129 Sv/J embryonic stem cells and backcrossed at least five times)	*Mop3*^*–/–*^*(BMAL1*^*–/–*^*)*	lower (insignificant) level of calcitonin	at 20 weeks of age: progressive weight loss	(21)
		significantly higher levels of osteocalcin	at 26 weeks of age: calcification in calcaneal tendon	
			at 35 weeks of age: severe bony ankylosis	
C57BL/6J	chronic jetlag	reduction in expression of FXR	at 16 weeks of age: hepatomegaly	(16)
	*Alb*^*cre*^*; BMAL1*^*fl/fl*^	increment in CK19, Cyp2B10, Cyp7A1 (Intrahepatic bile acid accumulation)	at 12–90 weeks of age: NAFLD, fibrosis, chronic liver inflammation, bile duct proliferation, hepatocyte proliferation	
	*Cry1*^*–/–*^*; Cry2*^*–/–*^	increment in SREBP and PPARγ (NAFLD)	at 42 weeks of age: HCC	
	*Per1*^*–/–*^*; Per2*^*–/–*^	P-β-catenin, c-Myc, p53, and Ki67(HCC)		
C57BL/6J × 129SvJ	jetlag	BMAL1 mutation augmented tumorigenesis and accelerated progression via a p53-dependent mechanism	increased tumor burden and decreased survival	(24)
	*Per2 (Per2*^*tm1Brd*^*/J)*	Per2 mutation exacerbated lung cancer progression via increment of c-Myc		
	*BMAL1 (ARNTL*^*tm1Bra*^*/J)*	Per2 mutation led to glucose and glutamine uptake		
	*BMAL1 (ARNTL*^*tm1Weit*^*/J)*			
human lung adenocarcinoma		ARNTL, CRY2, and PER3 expression decreased in grade 3 tumor		(24)
		increased c-Myc activity in tumors with lower PER2 expression		
human pancreatic ductal adenocarcinoma		low BMAL1 expression cases up to 70.1%	positive correlation of low BMAL1 expression and survival/disease-free survival times	(25)
		the ratio of low BMAL1 cases increased with advanced cancer stages		
human	pancreatic cancer patient	low BMAL1 mRNA and protein levels in tumor but high levels in noncancerous tissue of the same patient	BMAL1 was negatively correlated to the TNM stage	(26)
BxPC-3 pancreatic cancer cell line	shBMAL1	upregulated genes enriched in processes of cell division, mitosis, and cell cycle	increased cell proliferation and more colony formation	(26)
		downregulation in apoptotic-related genes	cell count increased in G0/G1 phase but reduced in G2/M phase	
		downregulated genes involved in p53, NF-κB, and PI3K-Akt pathway		
		downregulation in p-p53, p21, Bax, and Puma but upregulation in Cyclin B1, Bcl-2, and Bcl-xl		
AsPC-1 pancreatic cancer cell line	Lv-BMAL1 (BMAL1 overexpression)	cell arrested at G2/M phase	decreased cell proliferation	(26)
		upregulated in p-p52, p21, Bax, and Puma while reduction in expression of cyclin B1, Bcl-2, and Bcl-xl	reduced colony formation and cellular invasiveness	
nude mice	BxPC-3/shBMAL1	higher MMP-2 and MMP-9 levels in BxPC-3/shBMAL1-derived xenograft tumor tissue	smaller and lighter tumors derived from AsPC-1/Lv-BMAL1 cells	(26)
	AsPC-1/Lv-BMAL1			
mice (species not stated)	ARNTL^*pan-/–*^ (pancreatic-specific ARNTL-knockout mice)	increased HMGB1 expression in serum and pancreatic tissue	higher iron levels in the pancreas	(28)
	l-arginine-induced pancreatitis	reduced in mRNA expression of *Slc7a11, Gpx4, Sod1, Txn, Nfe2l2, Chmp5*	reduced animal survival	
		decreased in protein level of SLC7A11, GPX4, SOD1, TXN, NFE2L2, CHMP5	decreased serum amylase and pancreatic MPO activity	

Similar to circadian dysfunction by clock gene deletion or mutation, chronic circadian disruption such as chronic jetlag could potentially induce liver disease. As demonstrated by Kettner et al., both deletion of core clock genes (including *BMAL1*, *Per1/Per2*, or *Cry1/Cry2*) and chronic jetlag could induce hepatomegaly, nonalcoholic fatty liver disease (NAFLD), and even spontaneous hepatocellular carcinoma (HCC) with a sufficient induction period.^16^ The authors revealed that chronic circadian disruption could cause dysregulation of the sympathetic nervous system (SNS), which results in activation of the constitutive androstane receptor (CAR) and inhibition of the Farnesoid X receptor (FXR). The combined effect of intrahepatic bile acid accumulation and progression to nonalcoholic steatohepatitis (NASH) and advanced liver fibrosis activated by circadian disruption was the major cause of spontaneous HCC. In addition to hepatic diseases, the concept of circadian dysfunctions inducing obesity and leptin resistance were demonstrated by the same research team in the previous year. It has been revealed that both the central clock and the peripheral clock control leptin endocrine homeostasis. BMAL1/CLOCK in adipose tissue controls leptin transcription, driving the rhythmicity of serum leptin.^17^ Briefly, BMAL1/CLOCK could stimulate C/EBPα for leptin transcription at a particular phase, but chronic jetlag abolished the rhythmic bindings. Although the serum leptin levels were higher, failure to activate the pSTAT3/POMC pathway indicated leptin resistance induced by chronic jetlag.

Adiponectin and leptin are both recognized as family members of adipokines, and the secretion of leptin was elucidated to be controlled by the circadian clock. Similarly, in 2015, Barnea et al. suggested that the expression of adiponectin occurred in a circadian manner and its expression mediators, peroxisome proliferator-activated receptor γ (PPARγ) and PPARγ coactivator 1α (PGC1α), should be known as CCGs.^22^ It was found that expression of *AdipoQ* (encoding gene of adiponectin) is positively controlled by PPARγ+RXR and CLOCK+BMAL1 but negatively related to the addition of CRY1. Furthermore, the expression of *adipoQ* significantly reduced when the core clock gene *Clock* was silenced. However, the underlying molecular mechanism required further clarification. *Clock* is the first clock gene identified in vertebrates that is similar to *BMAL1*, which encodes the basic helix–loop–helix (bHLH)-PAS transcription factor. Early in the year 2000, Oishi et al. proved that *BMAL1* loses its rhythmicity and is sharply expressed at all phases in the brain, liver, heart, and kidney when the *Clock* is mutated, while at the same time expression of *Per2* and *Dbp* dramatically decreases without significant oscillation.^23^

#### 1.2.2 Role of BMAL1 in Cancer Progression

It has been found that the effects of circadian disruption are not limited to metabolic disease but also contribute to cancer progression (Table 1). In 2016, Papagianakopoulos et al. demonstrated that lung cancer progression was promoted and accelerated by jetlag.^24^ Both *Kras* and *Kras*^*+p53*^ mutations contributed to spontaneous tumorigenesis in mice, but it was found that jetlag induction during the tumor progression period significantly increased the tumor burden. To dissect the role of clock genes in tumorigenesis, *Per2* and *BMAL1* mutations were assessed. The finding showed that the loss of BMAL1 and Per2 further increased tumor burden and decreased survival, which comparatively had a greater impact in the *BMAL1*-mutated group. Furthermore, it was revealed that the *BMAL1* mutation could increase the tumor burden, possibly via a p53-dependent mechanism, while the *Per2* mutation was related to an increase in c-Myc. Lastly, an increase in lung cancer proliferation was observed to be associated with the consumption of glucose and glutamine, implying that cancer progression could be highly related to metabolism controlled by circadian genes.

One study suggested that human pancreatic ductal adenocarcinoma could be predicted by BMAL1 expression. According to Li et al. (2016), up to 70.1% of patients with pancreatic ductal adenocarcinoma exhibited low expression of BMAL1 in tumor samples. Among them, the cases could be further classified into clinical stages I, II, III, and IV with percentages of 30.8%, 61.3%, 84.6%, and 94.1%, respectively, which indicated the ratio increased with advancing stages and positively correlated to overall survival and disease-free survival times.^25^ Another clinical case study by Jiang et al. confirmed the finding. The mRNA and protein levels of *BMAL1* were significantly lower in the pancreatic tumor as compared to the noncancerous tissue from the individual in up to 45 cases, and the expression was negatively correlated to the TNM stage.^26^ To determine the role of BMAL1 in pancreatic cancer, PC cell line BxPC-3 with a higher BMAL1 protein level was silenced with shBMAL1, while AsPC-1 with lower *BMAL1* expression was transfected to overexpress *BMAL1*. Surprisingly, upregulated genes of BxPC-3/shBMAL1 cells were primarily involved in survival and cell proliferation, while those related to the apoptotic process were dramatically downregulated in the GO analysis. Furthermore, it was revealed that genes associated with the p53 pathway, NF-κB pathway, and PI3K-Akt pathways were downregulated, indicating the suppressive role of BMAL1 in pancreatic cancer. Conversely, AsPC-1 that stably overexpressed *BMAL1* markedly decreased proliferation and arrested in the G2/M phase compared to its controlled counterpart. The tumor suppressive effect was maintained in xenograft models, resulting in a smaller tumor size and reduction in invasiveness when *BMAL1* was overexpressed. Most importantly, *BMAL1* was suggested to directly bind on the promoter region for transcription of the *p53* gene.

In line with the above findings, Kiessling et al. suggested tumor growth could be inhibited by enhancing the function of the circadian clock. It was found that by treating cancer cells with dexamethasone (DEX), an agonist of the glucocorticoid receptor, the rhythmicity of cells could be observed, accompanied by cell cycle regulators.^27^ Interestingly, after being treated with DEX, fewer cells entered in the S phase, while more were in the G0/G1 phase, which indicated a decrement in the number of cells undergoing DNA replication. DEX-induced activation of the circadian clock was assumed to be responsible for slowing tumor cell proliferation and upregulation of rhythmic clock genes, including *Per1, Per2, Cry1*, and *Nr1d1*, along with the protein level of BMAL1. To further confirm that the inhibitory effect of DEX on tumor growth was due to activation of the clock, the *BMAL1* gene was knocked down; as expected, the relative tumor volume could no longer be suppressed and the rhythmicity of most BMAL1 target genes was disrupted.

The importance of BMAL1 was also noted in ferroptotic cancer cell-associated inflammation.^28^ Nuclear protein HMGB1 is the mediator triggering the inflammatory response, and it was demonstrated that HMGB1 expression induced by ferroptosis in acute pancreatitis could be regulated by BMAL1. BMAL1 could reverse the downregulation of antioxidant enzyme expression and proteins involved in the membrane repair system, resulting in a protective effect against tissue injury and a reduction in the HMGB1 level that is positively correlated with cancer-related inflammation.

Collectively, it has been corroborated that BMAL1 exhibits conspicuous and causal roles in the incidence of metabolic diseases and cancer progression. Moreover, the above findings suggest the potential to involve BMAL1 in tumor inhibition strategies. However, it could not be elucidated if circadian disruption induced by jetlag, social jetlag, or shift work could lead to severe consequences, such as clock gene mutation or deletion. Nevertheless, circadian misalignment or abnormal expression might deteriorate disease conditions or progression. On the other hand, the evidence showed that exposure to air pollution could lead to circadian toxicity.^29^ Some of the proteins involved in the xenobiotic detoxification process are also found to have a molecular link with the circadian clock. For instance, the mRNA level of aryl hydrocarbon receptor (*AhR*) and its downstream detoxification enzymes were found to be regulated by the *Clock* gene.^30^ Another study demonstrated that the inhibition or disruption of *Per1* and *Per2* might result in greater expression of *cyp1a1* and *cyp1b1* induced by TCDD in both in vivo and in vitro studies, which suggests an important role for *Per1* in AhR-regulated gene modulation.^31^ Neuronal PAS domain protein 2 (NPAS2), as the paralog of CLOCK, was identified as a regulator of hepatic CYP1A2 in both cell and animal models.^32^ Both AhR and CLOCK are class I basic helix—loop–helix/Per-ARNT-SIM (bHLH-PAS) proteins that sense environmental signals and form heterodimers with class II proteins, resulting in target gene regulation.^33^ Huang et al. demonstrated high coordination of CYP1A1 and PER2 in the posterior pituitaries and livers of rats, which suggests the possible interaction or similar responses of AhR/ARNT and CLOCK/BMAL1 systems toward environmental changes.^34^ These studies hint at a causal loop between xenobiotic detoxification and circadian regulation. Therefore, this Review will be focused on the effect of food-borne activators of xenobiotic detoxification and their possible effect on circadian disruption.

## 2 AhR Activation Interferes with Circadian Rhythm

Several studies mentioned the interference in the circadian rhythm caused by environmental toxicants. For example, benzo[*a*]pyrene (B[*a*]P) may cause lung inflammation via its alteration of the circadian pattern of blood pressure.^35^ It has been reviewed that exposure to air pollutants may affect rhythmic pulmonary and cardiovascular functions and consequently increase the risk of vascular and cardiometabolic disorders.^29^ Another study suggested that parental DNA damage and hypermethylation of the *Per1* promoter caused by B[*a*]P could pass to offspring.^36^ B[*a*]P is a potent substrate for CYP1A1 and 1B1, and AhR is a ligand-activated transcription factor that can be activated by B[*a*]P and is responsible for the transcription of the genes involved in xenobiotic metabolism.^37^ However, further clarification is needed to understand the underlying mechanisms of these toxicants disrupting or destroying the circadian rhythm and causing disease.

### 2.1 ARNT and BMAL1

ARNT forms a heterodimer with activated AhR and subsequently regulates transcription.^38^ BMAL1 is a protein that has a sequence homology similar to the ARNT protein, which includes similar splice patterns of intron/exon and five conserved exons that compose the Per-ARNT-Sim (PAS) domain.^39^ Core clock genes, such as BMAL1, CLOCK, and the family members of Cry and Per, are known as PAS-domain-containing proteins, and the domain facilitates the formation of heterodimers for protein–protein interactions and self-regulation. Due to the similarity of PAS domains, activated AhR can form a heterodimer with BMAL1 instead of ARNT, which may consequently disrupt CLOCK/BMAL1 activation and dampen the transcriptional rhythm.

The ligands of AhR vary from environmental toxicants to pharmaceuticals and phytochemicals. Environmental toxicants include tetrachlorodibenzo-*p*-dioxin (TCDD), which has a high affinity to AhR, halogenated aromatics, and polyaromatci hydrocarbons (PAHs).^40^ Pharmaceuticals and phytochemicals that can act as AhR ligands can be classified by their sources, which may include dietary ligands such as quercetin and resveratrol, metabolized food components such as indole[3,2-*b*] carbazoles, and gut microbial metabolites such as butyrate and indole-3-acetic.^41^ In 2008, it was demonstrated that exposure to TCDD, an AhR agonist, could lead to changes in the gene expression of *BMAL1* and *Per1*.^42^ It was further proven by immunoprecipitation that heterodimerization of BMAL1 with AhR increased in mouse ovarian extract following TCDD intervention.^43^ This finding was also supported by a study that demonstrated the enhancement or alteration of circadian and clock-controlled metabolic genes in AhR depletion mice.^44^ Exposure to TCDD has also been found to abolish the oscillating expression of up to 5636 clock-controlled genes in the liver. The finding suggested that activation of AhR could severely disrupt the circadian regulation of hepatic metabolism.^45^ Supportingly, the binding of activated AhR to the E-Box of the promoters of the clock gene and lipolysis gene was found to increase and consequently lead to an alteration in adipose tissue rhythmicity and impairment of lipolysis in mouse adipose tissue.^46^ Transcription of *Per1* is driven by the binding of the CLOCK/BMAL1 heterodimer to the E-box of the Per1 promoter, and it was found that AhR activation could lead to dysregulation of rhythmic Per1 and contribute to metabolic issues.^47^

The findings of these studies suggest that activation of AhR can have a circadian influence that may subsequently affect the host metabolism. On the other hand, it was also reported that the timing of exposure to the pollutants or toxicants known as AhR ligands can be critical because of the responsive expression of the xenobiotic enzymes that are controlled by circadian regulation.^48^ Therefore, it should be understood that environment toxicants, including food-borne toxicants, could be potential nonphotic entrainments causing circadian disruption.

## 3 Polycyclic Aromatic Hydrocarbons (PAHs)

**Figure 3:**
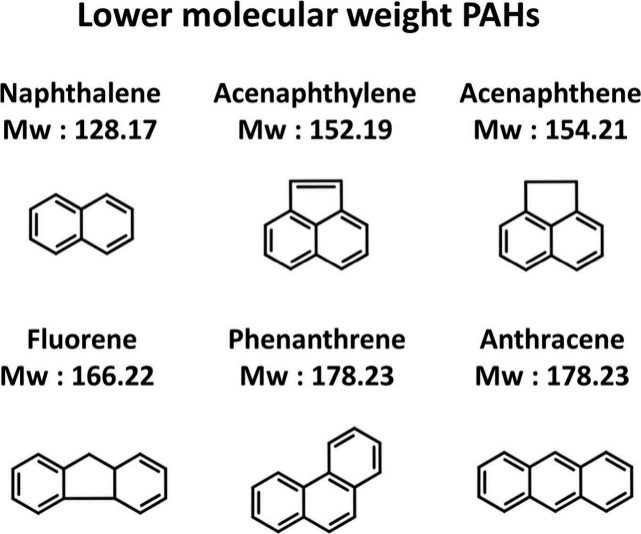
Lower molecular weight PAHs (2–3 rings).

**Figure 4:**
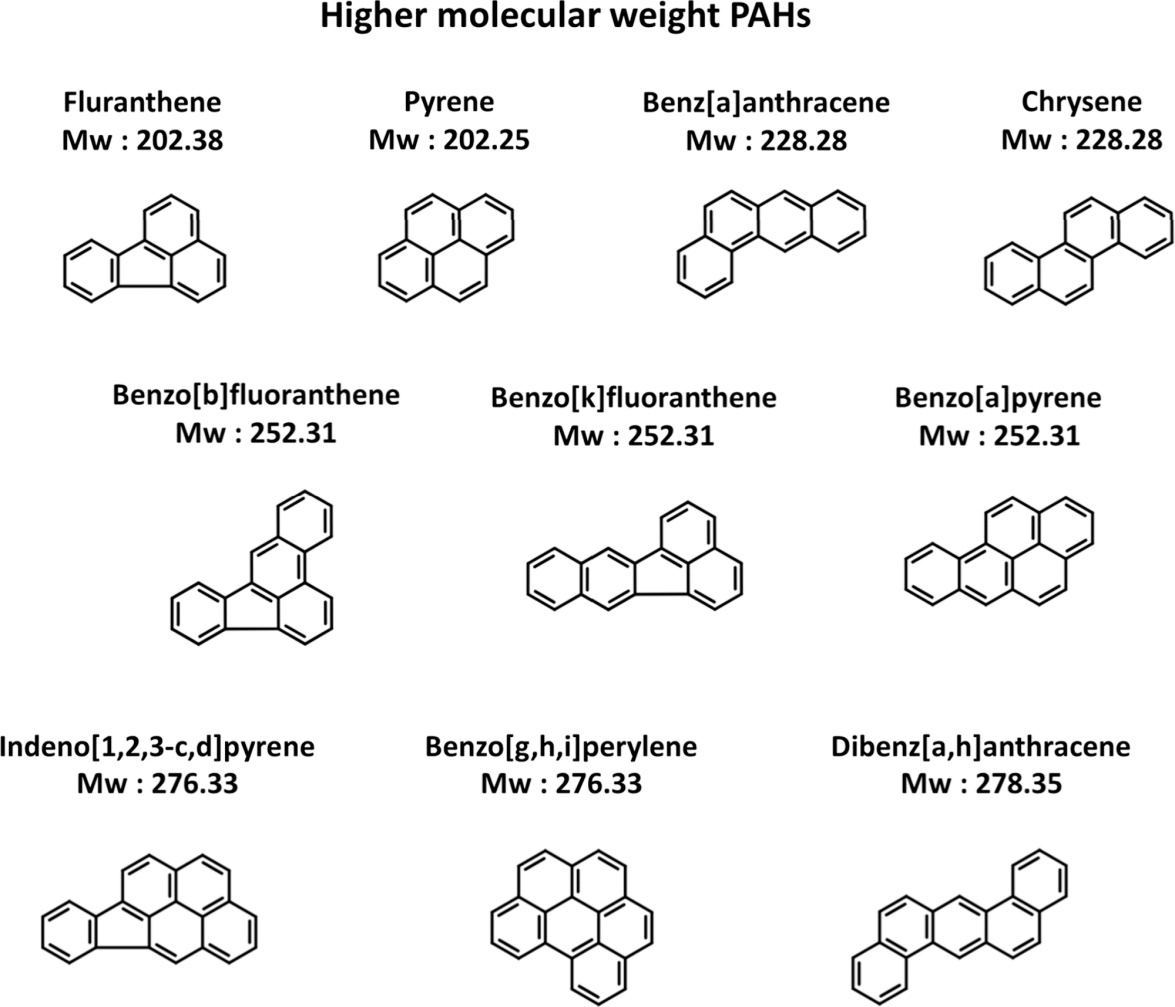
Higher molecular weight PAHs (more than 4 rings).

Polycyclic aromatic hydrocarbons (PAHs) are persistent environmental pollutants. Due to their ubiquity, actions for remedying the health issues caused by PAHs are a global concern.^49^ PAHs consist of multiple aromatic rings that can present in different molecular arrangements (linear, cluster, or angular) and are classified into different groups according to the number of rings present in the compounds, where those with lower molecular weight (2–3 rings) possess higher solubility in water (Figures 3 and 4).^50^ More than 200 PAHs can be generated via pyrolysis, a process that involves three main factors: organic matter, reduced oxygen levels, and high-temperature conditions. Human exposure to PAHs can occur through dietary and nondietary sources. The latter includes skin contact and inhalation, while the former can occur via the consumption of environmentally contaminated foods or foods that have undergone high-temperature processes, such as roasting, toasting, and frying.^51^

Previous studies have been conducted to determine the health effects of PAH exposure. A variety of adverse effects and health risks have been discovered, including carcinogenicity, teratogenicity, genotoxicity, neurotoxicity, and immunotoxicity. Short-term but high-concentration exposure can lead to skin and eye irritation, confusion, nausea, and impairment in lung function causing asthma, while long-term lower-dose exposure is implicated in the incidence of cancers, such as lung cancer, skin cancer, and cancers of the digestive tract.^52^ In addition, the association between long-term exposure to PAHs and an increase in the prevalence of hypertension has been reported in several studies due to the overactivation of AhR.^53^ Therefore, reducing opportunities for PAH exposure has become a major concern to protect public health and safety. Some strategies have been suggested to minimize the exposure risk of humans to PAHs, such as regulation of cooking or food processing practices.^54^ Nevertheless, once PAH contamination has occurred it is difficult remove^55^ and therefore methods for the detection of PAHs are required.

Besides inhaled air, dietary intake serves as a major route of human exposure to PAHs,^56^ and because it is controllable the detection of PAH in food has gained its place in the research field. For the detection of PAH contamination in food, common analytical targets include naphthalene (NA), anthracene (ANT), benzo[*a*]anthracene (BAA), dibenzopyrene (DBP), and benzo[*k*]fluoranthene (BKF).^57^ Among a list of PAHs that are commonly found in foods, 15 have confirmed genotoxicity.^57^ Until 2008, benzo[*a*]pyrene (B[*a*]P) was the only marker recognized for the identification of the occurrence of PAH contamination in food, however, several markers are currently used.^58^ The regulations were changed for the following reasons: (1) even if the concentration of B[*a*]P is low in food, contamination with other PAHs may still be present and (2) B[*a*]P is a minor contributor to total cancer risk at around 11%.^59^

Before introducing PAH2, PAH4, and PAH8 as more accurate markers, it is important to note that according to regulations, smoked molluscs, muscle meat of smoked fish, heat-treated meat products, cocoa butter, and chocolates are food commodities with high potential to be contaminated by B[*a*]P. The maximum allowable level of B[*a*]P is 5 μg/kg. Due to the contamination of PAHs in water, direct consumption of aquatic organisms could contribute to the intake of PAHs. Additionally, PAH contamination has been found in other foods, including wheat flour (total PAHs 0.71–1.66 μg/kg), toasted bread (total PAHs 7.38–18.0 μg/kg), cabbage (total PAHs 9 μg/kg), lettuce (total PAHs 14 μg/kg), and parsley (total PAHs 15–120 μg/kg). It should be noted that the contamination levels could be higher in industrial cities.^57^ Furthermore, meat products, oils, and water-based food products are also highly susceptible to PAH contamination. According to a review by Domingo and Nadal, the estimated intake of PAHs could reach up to 59.2 μg/day, although most studies have reported much lower intake amounts of PAHs.^60^

PAH8 includes benzo[*a*]anthracene (BAA), chrysene, benzo[*b*]fluoranthene, benzo[*a*]pyrene (B[*a*]P), benzo[*k*]fluoranthene, dibenzo[*a*,*h*]anthracene, benzo[*g*,*h*,*i*]perylene, and indeno[1,2,3-*c*,*d*]pyrene. These PAHs are considered potential carcinogens.^61^ PAH2 consists of chrysene and B[*a*]P, while PAH4 includes BAA, chrysene, benzo[*b*]fluoranthene, and B[*a*]P. Among them, BAA and chrysene are considered light compounds that can be easily inhaled.^62^ It was previously reported that up to 60% of lung cancers were caused by B[*a*]P and other PAHs found in cigarettes, with each cigarette potentially containing 20–40 ng of B[*a*]P. The carcinogenic response could also occur in the bladder, breast, cervix, and prostate.^63^ According to a recent clinical study investigating the correlation between smoking and oxidative stress in chronic obstructive pulmonary disease patients, B[*a*]P was the most abundant carcinogenic PAH in smokers and was positively correlated with higher serum uric acid levels.^64^ Although lower molecular weight PAHs are predominant in commercial cigarettes, B[*a*]P is used as a representative marker for total PAHs based on the strong positive correlation between them.^65^ However, B[*a*]P might not be an appropriate surrogate for all 14 PAHs in cigarettes.^66^ A multiyear study of PAHs in cigarettes suggested that the B[*a*]P level is representative of 4- and 5-ring PAHs, while fluorene is representative of 3-ring PAHs, with a high observed correlation.^67^

The toxicity of these PAHs was tested by Brunström et al., and the results showed that benzo[*k*]fluoranthene had the highest potency (LD_50_ of 56 nmol/kg egg), followed by dibenzo[*a*,*h*]anthracene (LD_50_ of 140 nmol/kg egg).^68^ In addition, dibenzo[*a*,*h*]anthracene and indeno[1,2,3-*c*,*d*]pyrene were found to have the highest potency in inducing EROD enzyme activities. Indeno[1,2,3-*c*,*d*]pyrene was found to be a prominent PAH in ambient PM2.5, which could be responsible for enhancing antigen-induced allergic inflammatory responses.^69^ In 2005, it was demonstrated that exposure to benzo[*k*]fluoranthene could reduce splenic and thymic cellularity in mice.^70^ Moreover, exposure to benzo[*k*]fluoranthene could lead to genotoxic damage by inducing oxidative stress, as evidenced in an in vivo study.^71^

Although B[*a*]P was declared inappropriate as the marker for the occurrence of PAH contamination in food, it remains the most extensively studied PAH due to its high toxic equivalency factor (TEF) value and genotoxicity.^57^ Because B[*a*]P is the most well-studied food-borne PAH and serves as one of the most famous ligands of AhR, we focus on it in the following section.

### 3.1 Benzo[*a*]pyrene (B[*a*]P)

As previously described, B[*a*]P is a highly toxic carcinogenic PAH. Up to 95% of nonsmokers’ B[*a*]P exposure is from diet.^72^ The International Agency for Research on Cancer (IARC) has designated B[*a*]P as a group I human carcinogen, and this classification is supported by evidence from preclinical and epidemiological studies.^73^ B[*a*]P exerts its genotoxic effects following metabolization by cytochrome P450 (CYP) enzymes. In detail, B[*a*]P is converted into BaP-7,8-oxide by CYPs, then converted into dihydrodiol by microsomal epoxide hydrolase (mEH), and consequently becomes B[*a*]P-7,8-dihyrodiol-9,10-epoxide (BPDE) within this three-stage formation pathway.^74^ BPDE is the active metabolite that reacts with DNA directly and forms an adduct, preferentially a premutagenic guanine adduct, which finalizes in G to T transversion.^75^

#### 3.1.1 Phase I Enzymes

CYPs were first discovered by Klingenberg in 1954, and their function and importance were determined nearly a decade later. Numerous CYPs are involved in drug metabolism or xenobiotic detoxification metabolism.^76^ The involvement of the CYP450 enzyme is compulsory in the detoxification process to mediate phase I reactions, including oxidation, reduction, or hydrolysis reactions that result in metabolized molecules with an introduced functional group (−OH, – SH, – NH_2_, or – COOH).^77^ In the metabolism of B[*a*]P, the isoforms CYP1A1 and CYP1B1 are responsible for the production of epoxide intermediates, including B[*a*]P-7,8-oxide, B[*a*]P-9,10-oxide, or 3-hydroxy-B[*a*]P, and the conversions by CYP450 enzymes are known to be microsomal NADPH-dependent.^78^ Although there are 18 mammalian CYP families, families CYP1–4 are responsive and inducible by environmental factors, such as diet, drugs, and chemical inducers.^79^ It was previously reported that PAHs can induce several different members of CYP families, and B[*a*]P is a potent substrate for CYP1A1 and 1B1.^80^ The efficiency of CYP1B1 to oxidize B[*a*]P is around half that of CYP1A1, and it is notable that other family members, including CYP1A2, CYP2C8, CYP2C9, and CYP3A4, have also been found to be involved in B[*a*]P oxidation.^81^

Oxidation of B[*a*]P by CYP enzymes is followed by the catalysis of epoxide hydrolases (EHs) to promote the biotransformation of highly reactive epoxides into diols.^82^ These hydrated products include B[*a*]P-9,10-diol from B[*a*]P-9,10-oxide and B[*a*]P-7,8-diol from B[*a*]P-7,8-oxide. At this stage, metabolic activation by phase I enzymes leads to the formation of carcinogenic metabolites, while the involvement of phase II enzymes leads to a detoxification pathway.^83^ B[*a*]P-7,8-diol is mono-oxygenated by CYP1A1 and CYP1B1 and finally yields B[*a*]P-7,8-diol-9,10-epoxide (BPDE), the electrophilic carcinogen that can covalently bind to DNA guanine residues to form adducts.^78^ As a reactive electrophile, the formation of BPDE can cause DNA damage, DNA mutations, and carcinogenesis. High levels of BPDE-DNA adducts could even increase the risk of abortion during early pregnancy.^84^ According to previous studies, 0.01–0.1 μM BPDE could form 800–9600 bulky DNA adducts. The unrepaired BPDE-DNA adducts could lead to toxicity caused by replicative stress and genomic instability.^85^ In the in vitro study, a significant increase in the level of DNA lesions was detected upon treatment with BPDE at a lower concentration of 10 nM.^86^ Notably, BPDE can form adducts not only by binding to nucleic acids but also by binding to proteins and lipids.^87^ Although CYP450 enzymes are responsible for the formation of BPDE, their primary role and ultimate purpose are detoxification and protection of the host.

#### 3.1.2 Phase II and Phase III of the Detoxification Process

The first phase of the detoxification process by CYP450 enzymes is followed by phase II detoxification: the conjugation of endogenous hydrophilic substances at the reactive sites of phase I metabolites.^88^ The corresponding enzymes for the conjugation are known as phase II enzymes and include glucuronyl transferase, sulfotransferase (SULT), glutathione transferase, *N*-acetyl transferase, and methyltransferase for the transfer of glucuronic acid, sulfate, glutathione, an acetyl group, and a methyl group onto a phase I metabolite, respectively.^88^ UDP-glucuronosyltransferase (UGT), SULT, and glutathione *S*-transferase (GST) catalyze most conjugation reactions among these phase II enzymes.^77^ The conjugation of these anionic groups increases the hydrophilicity of the parent compound and stops these compounds from diffusing across the phospholipid membrane barrier.^89^ In each stage of the B[*a*]P detoxification process, different phase II enzymes are responsible for the formation of detoxified products. For instance, GSTs can catalyze the conjugation with B[*a*]P-7,8-epoxide to form glutathione conjugates, while UGTs and SULTs are involved in the conjugation with B[*a*]P-7,8-dihydrodiol.^90^

In the final stage, also called phase III, the metabolites with hydrophilic conjugates can be excreted or eliminated via membrane carriers, primarily ATP-binding cassette (ABC) and solute carrier (SLC) transporters.^91^

### 3.2 Aryl Hydrocarbon Receptor (AhR) Activation

**Figure 5:**
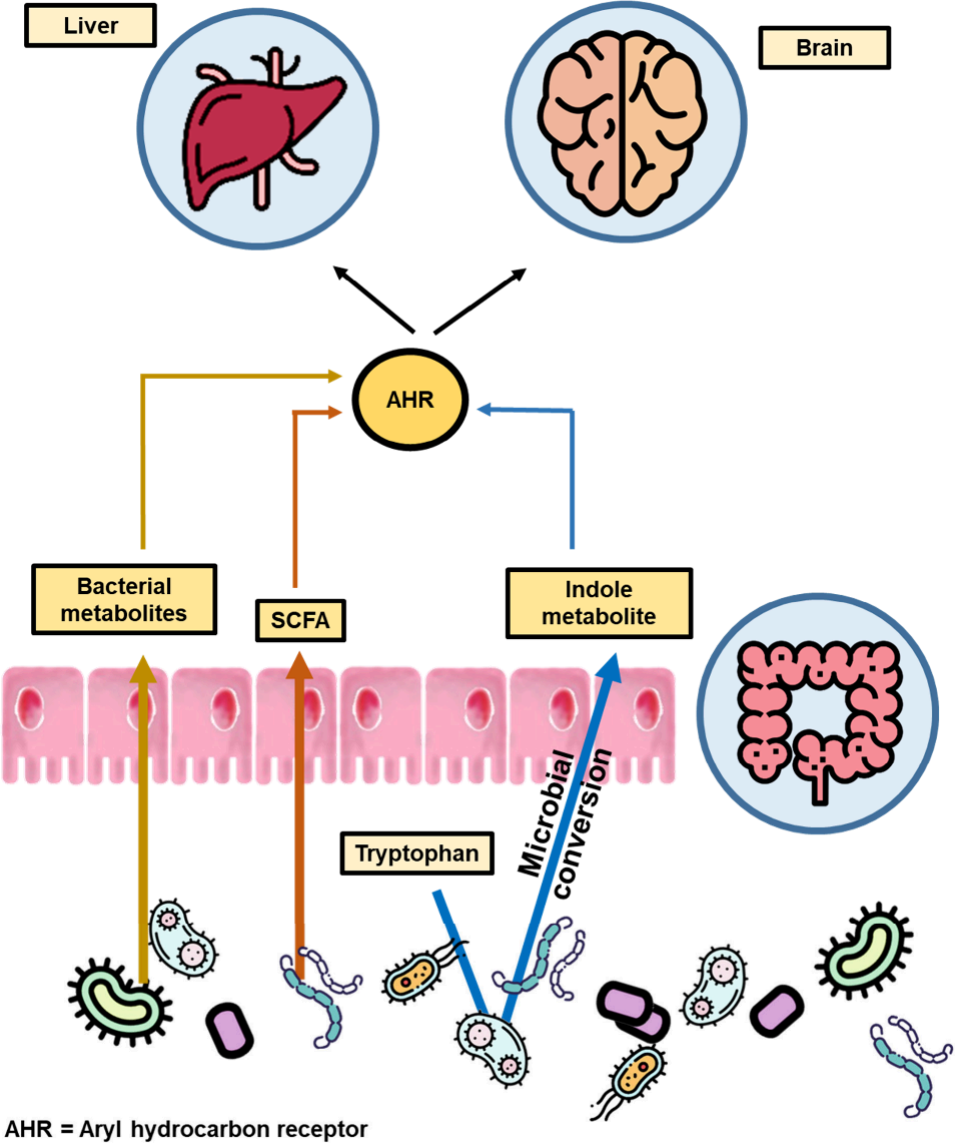
Some microbial metabolites are known AhR activators. Modulation of gut microbiota leads to the production of distinct gut microbial metabolites, including short-chain fatty acids and indoles. These metabolites subsequently affect the activation of certain receptors, such as AhR.

In addition to environmental toxicants, several AhR ligands have been identified previously, including endogenous ligands such as tryptophan metabolites and gut microbiota-derived compounds (Figure 5).^40^ On the AhR protein, there is a PAS-B domain that allows binding of ligand,s while the bHLH-PAS-A domains on both AhR and ARNT proteins allow their dimerization.^92^ For instance, the binding of B[*a*]P to AhR leads to the dimerization of AhR and aryl hydrocarbon receptor nuclear translocator (ARNT), followed by nuclear translocation and binding of the heterodimer onto DNA, such as xenobiotic responsive elements (XREs), resulting in the transcription of regulated genes, including CYP enzymes.^92,93^ Although xenobiotic metabolism is the first discovered function of AhR, its activation also controls several mechanisms including cell cycle regulation, protein interaction, and epigenetic mechanisms.^92^

It has been reported that oxidative stress via AhR activation by B[*a*]P could lead to mitochondria-mediated intrinsic apoptosis.^94^ Additionally, under conditions with abundant endogenous AhR levels, the pro-apoptogenic effect of B[*a*]P via CYP1A1 activation is enhanced.^95^ Another study suggested that oral gavage of B[*a*]P disrupts fat synthesis and glucose homeostasis and induces inflammation, possibly via AhR activation.^96^ Activation of AhR by B[*a*]P also led to pro-inflammatory responses in respiratory allergy via increased IL-33 expression and eosinophil infiltration in the lung.^97^ These studies suggest the deleterious effect on human health caused by B[*a*]P-activated AhR.

## 4 Intervention or Supplementation of Phytochemicals Potentially Improves Circadian Disruption

In medicine, phytochemicals have renowned advantages associated with treatment costs and alleviation of side effects and, at the same time, exhibit preventive and therapeutic effects. Several studies show the effectiveness of phytochemicals in disease attenuation or gut microbial dysbiosis improvement. In comparison, the effect of phytochemicals on circadian disruption is not as commonly recognized. In this section, some recent studies demonstrating the effect of phytochemicals on circadian rhythm disorders will be discussed.

In 2020, Song et al. reported that flavonoids from *Cyclocarya paliurus* showed modulatory effects on constant-darkness-induced mice in terms of their hepatic clock gene misalignment and intestinal microbiota dysbiosis.^98^ The extract was rich in kaempferol and quercetin derivatives, and the major outcomes of the supplementation were reflected in the body weight, the decrement of the F/B value, gut microbial diurnal oscillation, and the corresponding functionality of gut microbes. In the same year, it was found that polymethoxyflavones containing 3,5,7,3′,4′-pentamethoxyflavone, 5,7,4′-trimethoxyflavone, and 5,7-dimethoxyflavonein black ginger (*Kaempferia parviflora*) were regulative, reflected in the expression of CCGs including Bmal1, Cry1, and Per2.^99^ Moreover, the extract also ameliorated some negative effects induced by jetlag in mouse models. Qi et al. revealed that dietary tea polyphenols composed of gallic acid, epigallocatechin, epigallocatechin-3-gallate, and epicatechin-3-gallate could ameliorate memory impairment and metabolic disorder, including insulin resistance and glucose/lipid metabolism dysfunction induced by constant darkness in a mouse model.^100^ The same research team also found that tea polyphenols could mitigate circadian clock dysregulation induced by oxidative stress, particularly the mitochondria impairment elicited by hydrogen peroxide via Bmal1-dependent pathways, such as Nrf2/ARE/HO-1 and Akt/CREB/BDNF signaling pathways.^101^ Further studies have determined that dietary phytochemicals, such as dietary natural cocoa,^102^ nobiletin,^103^ myricetin,^104^ EGCG,^105^ resveratrol,^106^ pterostilbene,^107^ and caffeine,^108^ could potentially ameliorate circadian rhythm disruption or overcome health issues induced by circadian dysregulation. Therefore, dietary phytochemicals are potential modulatory agents for circadian rhythm disruption or sleep disorders.

On the other hand, phytochemicals or plant compounds with antioxidant properties can also protect the circadian clock by directly reducing the formation of PAHs in food. The pyrolytic formation of PAHs is a crucial process that generates a significant amount of free radicals. These radicals may ultimately form aromatic hydrocarbon rings. For instance, methyl radicals and acetylene radicals can form benzene rings. Additionally, small radical molecules can form aromatic hydrocarbon rings through polymerization, addition reactions, and cyclization reactions.^109^ Additionally, it was reported that edible oils account for approximately 50% of dietary exposure to PAHs, possibly due to the high oil solubility of these PAHs.^110^ Several factors might contribute to the occurrence of PAHs, such as the types of raw materials used for edible oils, refining processes, storage conditions, and the types of cooking oils.^111^ The production of cyclohexane or hydroperoxides occurs during the thermal treatment of food lipids through oxidation and breakdown. This process ultimately forms naphthalene or naphthalene-like compounds through further oxidation or cyclization and may result in the synthesis of PAHs through polymerization. In another context, exposure of B[*a*]P to UVA radiation can cause ROS accumulation in lipids, and these concentrated ROS can induce lipid peroxidation.^112^ Although the formation of PAHs in oil is not well-verified, it is believed that they could form due to free radicals generated from fatty acid oxidation, which could be evidenced by the finding showing that catechin in oil can effectively inhibit the generation of PAH4 at levels above 0.02% under heating conditions.^113^ Additionally, more studies have demonstrated that nonlipid substances with antioxidant properties in oil can significantly inhibit PAH formation during heating processes.^114^ The inhibition of PAH formation, accompanied by the reduction in the peroxide value (POV) and thiobarbituric acid reactive substances (TBARs) by apple polyphenols in barbecued pork, suggested the contribution of lipid oxidation to PAH formation.^115^ The use of rapeseed oil, sesame oil, or sunflower oil could help reduce PAH formation.^116^

Therefore, eliminating radicals with antioxidants could be a potential strategy. It has been suggested that supplementing phytochemicals with antioxidant properties could achieve this goal. For instance, the formation of PAHs during the roasting process of sunflower seeds could be significantly reduced when the seeds were flavored with hogweed, which contains several phenolic compounds with strong antioxidant properties, such as rutin, coumarin, and quercetin.^117^ Another study suggested that a marinade containing dietary antioxidants, including quercetin or the volatile organosulfur phytochemical in garlic, diallyl disulfide, could effectively decrease PAH levels in grilled meat.^118^ As mentioned above, PAH8 has been introduced as a more accurate marker to indicate the contamination of PAHs in food. As demonstrated by Wang et al., naringenin and quinic acid exhibited a compelling inhibitory effect on PAH8 formation in charcoal-grilled chicken wings.^119^ Although the authors suggested that there was no correlation between the reduction of phenolic compounds and PAH8 production, more antioxidant analysis should be conducted before a conclusion is reached. In another study, it was suggested that ABTS could effectively demonstrate the correlation between the antioxidant properties of flavonoids and PAH formation.^120^ This indicates that the antioxidant properties of phytochemicals could be indicative of their capability to inhibit PAH formation.

There are several mechanisms that have been pointed out to ameliorate circadian misalignment, but the involvement of AhR regulation was not mentioned.

## 5 Perspective: Effect of Phytochemicals on AhR Activation

Due to the crucial role of AhR in circadian regulation, modulating the activation of AhR represents a strategy to prevent consequential disruption. Some phytochemicals may act as potential contributors. For instance, resveratrol has been reported to possess antagonistic activity on AhR to compete with TCDD, as resveratrol has a close structural homology to AhR antagonistic ligands.^121^ Although resveratrol was not as efficient as an AhR inhibitor compared to α-naphthoflavone, it has the advantage of lower toxicity. Human paraoxonase 1 (PON-1) is a hepatic-secreted enzyme that is transcriptionally regulated by AhR. Interestingly, compared to quercetin, TCDD is a poor inducer in regulating the gene expression of PON-1.^122^ These results suggested that the binding of different AhR ligands may lead to distinct gene regulation depending on the target sequence. Therefore, the intervention of phytochemicals to compete with environmental toxicants upon AhR activation could be a possible strategy to avoid circadian disruption while at the same time maintaining xenobiotic metabolism.

### 5.1 From Flavonoids to Stilbenoids

The role of phytochemicals in AhR activation can be agonistic or antagonistic. It has been reviewed that flavonoids that share a similar structural backbone with quercetin, such as daidzein, genistein, and apigenin, may act as AhR agonists, but at the same time some may also exhibit antagonistic activity to inhibit the activation of AhR induced by environmental pollutants.^123^ For example, one flavonoid that can be naturally found in citrus peel was reported to reduce the nuclear translocation of AhR induced by 7,12-dimethylbenz[*a*]anthracene (DMBA) or TCDD, as well as its downstream gene expression.^124^ A molecular docking study screened a list of flavonoids and showed that some could possess blocking capacities for AhR-ARNT heterodimer formation up to 50–60%, while most of the studied flavonoids interact with AhR and ARNT to some extent.^125^ Notably, fisetin showed the greatest blocking capability among the studied flavonoids, and more importantly resveratrol, a member of the stilbene family, also showed a probability of blockage of 30%, suggesting the potential of stilbenoids to suppress the transcriptional ability of AhR.^125^

Chronic activation of AhR has been found to promote the expression of resistant genes to the inhibitor of cutaneous melanoma, which may subsequently lead to aggressive tumors. The addition of resveratrol in treatment with the inhibitor could effectively reduce the number of resistant cells by inhibiting AhR activation, delaying tumor growth.^126^ In 1999, resveratrol was revealed as a competitive antagonist of dioxin and other AhR ligands.^121^ Resveratrol intervention has also been reported to rescue osteoblastogenesis from deterioration induced by indoxyl sulfate, a gut microbial tryptophan metabolite, by acting as an AhR antagonist.^127^ Moreover, resveratrol could also prevent epigenetic silencing of the *BRCA-1* gene, which is AhR-dependent, and lead to the repair of DNA damage caused by xenobiotics.^128^

It was reviewed that the binding energies of resveratrol and its derived metabolite, lunularin, against the PAS-A domain of AhR were −6.64 and −6.76 kcal/mol, respectively.^129^ In 2010, piceatannol, hydroxylated resveratrol, was found to exhibit a similar antagonistic effect on AhR activation^130^ which indicates that the stilbenoids that share the same backbone are potential candidates as AhR antagonists. The inhibitory effect of pterostilbene, known as methoxylated resveratrol, on the nuclear translocation of AhR was reported to protect keratinocytes against the damage induced by particulate matter.^131^

**Figure 6:**
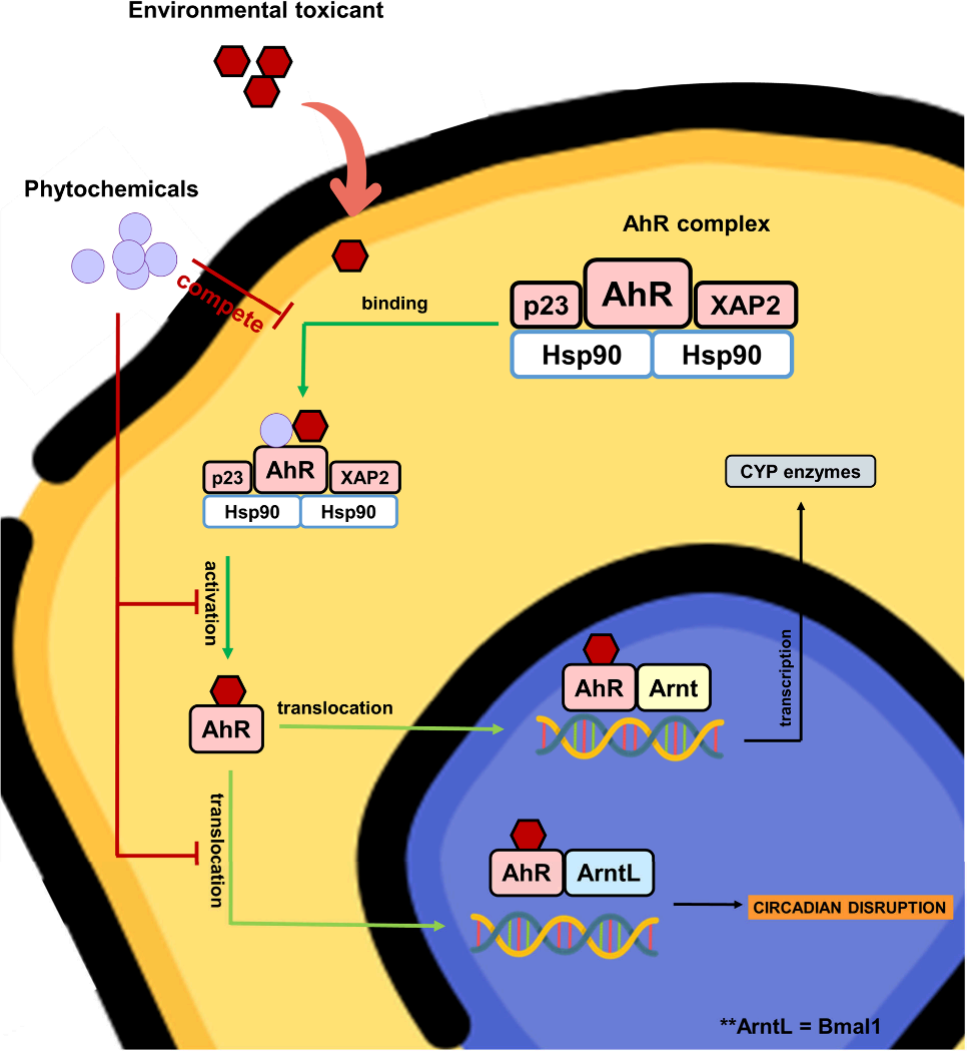
AhR activation by environmental toxicants may be responsible for phase I enzyme transcription and circadian disruption. Some phytochemicals may exhibit an antagonistic effect on AhR activation.

Environmental pollutants and food-borne pollutants have become unavoidable issues to be faced in this age. In addition to cancers and metabolic diseases, circadian disruption has also been known to be influenced by these pollutants via AhR activation. More importantly, circadian disruption has recently been reported to adversely affect the host metabolic function. Inhibition of excessive AhR activation has been identified as a strategy to alleviate the progression of the disease. On the other hand, some phytochemicals such as flavonoids and stilbenoids have also been found to exhibit an antagonistic effect on AhR activation (Figure 6). However, there are fewer studies on the suppressive effect of AhR activation against food-borne pollutants in circadian disruption. Hopefully, this review will provide new insights into preventing pollutant-induced circadian disruption via AhR overactivation through phytochemical intervention.
